# OP50, a bacterial strain conventionally used as food for laboratory maintenance of *C. elegans*, is a biofilm formation defective mutant

**DOI:** 10.17912/micropub.biology.000216

**Published:** 2020-02-05

**Authors:** Yukinobu Arata, Taku Oshima, Yusaku Ikeda, Hiroshi Kimura, Yasushi Sako

**Affiliations:** 1 Cellular Informatics Laboratory, RIKEN, 2-1 Wako, Saitama, 351-0198, Japan; 2 Department of Biotechnology, Toyama Prefectural University, 5180 Kurokawa, Imizu, Toyama 939-0398, Japan; 3 Department of Mechanical Engineering, School of Engineering, Tokai University, 4-1-1 Kitakaname, Hiratsuka, Kanagawa 259-1292, Japan

**Figure 1:**
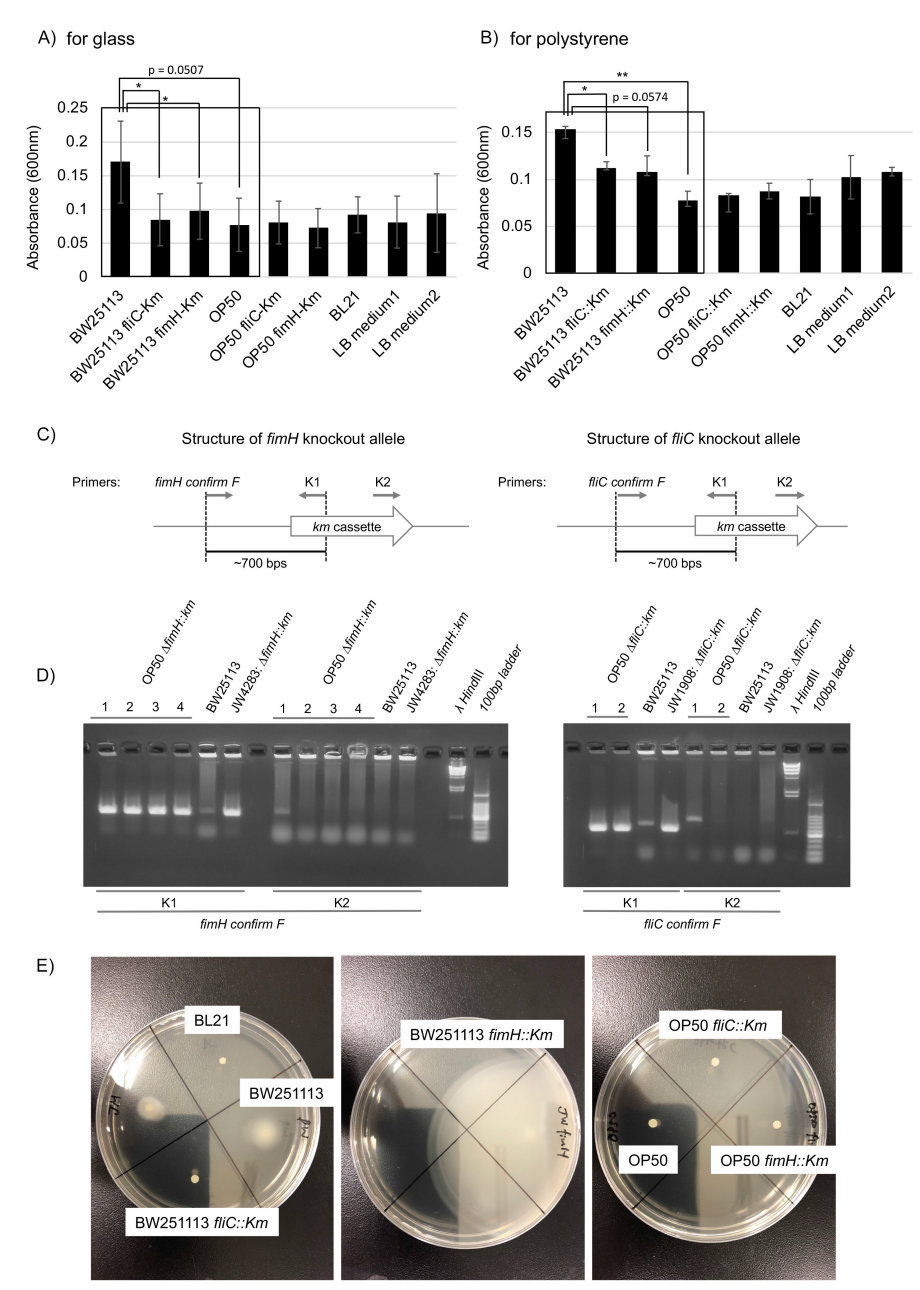
**Biofilm formation, the establishment of OP50-derived *fliC* and *fimH* knockout strains, and bacterial motility assay.** Biofilm formation on glass (A) and polystyrene (B). Biofilm formed by indicated *E. coli* strains on glass and polystyrene quantified by CV staining. Optical absorbance at 600 nm was measured in three independent experiments and compared with paired student t-tests; *p < 0.05, ** p < 0.005. Biofilm formation ability of OP50 *fliC::Km*, OP50 *fimH::Km*, and BL21 should be studied further with rigorous experiments and statistical testing. (C and D) Isolation of OP50 *fimH::Km* and OP50 *fliC::Km*. (C) Schematic structure of *fliC* and *fimH* knockout alleles with position and direction of primers for PCR. *Km* cassette was integrated in gene locus to form *fimH::Km* and *fliC::Km.* (D) Confirmation of the presence of *fimH::Km* and *fliC::Km* in OP50-derived *E. coli* colonies via P1 transduction. Bacteria obtained from Km-resistant colonies were subjected to PCR with the indicated primers. DNA fragments of expected sizes from the genomic structure of the *fliC::Km* and *fimH::Km* knockout alleles were amplified with a primer set with K1 but not K2 primer. (E) Motility of *E. coli* strains. Motility of indicated *E. coli* strains was compared by colony size after cultivation on soft agar LB plates.

## Description

OP50 is an *Escherichia coli* strain conventionally used as a bacterial food in the laboratory maintenance of *Caenorhabditis elegans* on agar plates. It has also been used to feed *C. elegans* in longitudinal cultures within microfluidic devices (MFDs) (Hulme *et al.*, 2010; Li *et al*., 2015), where it has been subject to killing by ultraviolet irradiation or pasteurization performed to suppress clogging due to biofilm formation and aggregation (Li *et al.*, 2015; Zhuo *et al.*, 2017). However, the killed bacterial food can change *C. elegans* aging dynamics, likely due to influences on *C. elegans* physiology (Saul *et al.*, 2009; Gruber *et al.;*, 2007; Garigan *et al.*, 2002). Further development of longitudinal culturing systems for *C. elegans* in MFDs requires elucidation of the mechanisms that underlie food bacteria clogging and delineation of culture conditions in which living bacterial food can be incorporated without clogging. Bacteria switch from planktonic growth to aggregated growth under conditions of environmental stress, in the presence of toxins (e.g. antibiotics), and when there is a lack of nutrients (Trunk *et al.*, 2018). Biofilms, such as dental plaque, are bacterial communities that are organized in a film-like form in which they are embedded in a self-produced polymeric matrix on biotic or abiotic surfaces; pellicles are floating biofilms that form at liquid-air interfaces. Meanwhile, autoaggregations are aggregated communities of bacteria suspended in solution, such as bacterial flocs formed in activated sludge. Biofilms and autoaggregations are formed by both shared and independent genetic and physico-chemical mechanisms (Trunk *et al.*, 2018; Berne *et al.*, 2018; Berne *et al.*, 2015). In this study, we examined OP50 biofilm formation.

Biofilm formation is mediated by flagellin proteins (e.g. FliC), which form flagella, and the adhesion protein FimH, which is located at the tips of type I pili (Berne *et al.*, 2018, Jones et al., 1995; Pratt and Kolter, 1998; Friedlander *et al*., 2013). We compared the biofilm formation ability of OP50 with that of the biofilm-forming (Wood *et al.*, 2006) wild-type BW251113 *E. coli* strain as well as that of two BW251113-derived knockouts produced with a kanamycin (Km) cassette characterized as biofilm formation defective mutants: JW4283: BW25113 *fimH::Km* (a *fimH* knockout) and JW1908: BW25113 *fliC*::*Km* (a *fliC* knockout) (Baba *et al.*, 2006). Compared to the original BW251113 strain, BW251113 *fliC*::*Km* had a significantlyreduced ability to form biofilm on glass and polystyrene (Fig. 1A and 1B, p < 0.05) and BW25113 *fimH::Km* had a significantly reduced ability to form biofilm on glass (Fig. 1A, p < 0.05; biofilm formation on polystyrene showed a near-significant reduction trend Fig. 1B, p = 0.0574). Compared with the original BW251113 strain, we found that OP50 had a significantly reduced biofilm formation ability on polystyrene (Fig. 1B, p < 0.05; biofilm formation on glass showed a near-significant reduction trend, Fig. 1A, p = 0.0507). The biofilm formation ability of OP50 was as low as that seen with the BW251113 biofilm formation defective mutants, and similar to that of OP50 *fliC::Km* and OP50 *fimH::Km* mutants (Fig. 1A and 1B), which were constructed by transferring *fliC::Km* and *fimH::Km* alleles to OP50 by P1 transduction (Fig. 1C and 1D). Therefore, we conclude that the original OP50 strain is itself a biofilm formation defective mutant.

Bacterial motility is required for biofilm formation. Despite of the presence of flagella, mutants of a motor protein that enables flagellar rotation have been shown to have a reduced biofilm formation ability (Pratt and Kolter, 1998; Friedlander *et al*., 2013; Wood *et al*., 2006). To study the mechanism of impaired biofilm formation ability of OP50, we performed a motility assay that showed that OP50 exhibited much lower motility than BW251113 (Fig. 1E). Motility was similar among the original OP50, OP50 *fliC::Km*, and OP50 *fimH::Km* strains (Fig. 1E). Preliminary mapping of a draft genome sequence of OP50 to that of a reference *E. coli* strain (REL606) identified non-synonymous nucleic acid substitutions in flagellar hook protein E, flagellar P-ring protein precursor I, and flagellar hook-associated protein K (personal communication with Robin C. May at University of Birmingham) (see Excel file in WormBase, ftp://ftp.wormbase.org/pub/wormbase/species/e_coli/op50/annotation/op50_annotated_snps.xls). These results suggest that OP50’s impaired biofilm formation is likely caused by a lack of functional flagella due to these point mutations on flagella genes.

In our experiments, the motility and biofilm formation ability on glass and polystyrene of BL21 strain *E. coli* was similar to that seen with BW25113 *fliC*::*Km* (Fig. 1A, 1B, and 1E). Our preliminary experiments suggest that BL21 is also a biofilm formation defective mutant with impaired motility. Impaired biofilm formation in BL21 and OP50 due to impaired motility is not necessarily caused by maintenance in nutrient-rich laboratory conditions, where bacteria can survive without active chemotaxis by flagella. It has been reported that flagellar regulon is frequently deleted secondarily to experimental genetic manipulation (Hobman *et al.*, 2007). Additionally, *E. coli* mutants with no functional flagella are selected in mouse intestine under certain conditions (Leatham *et al*., 2005). These phenomena are thought to be caused by high selective pressure from extracellular nutrient conditions on the flagellum regulon, because flagella are high energy consumers. Given that non-flagellar mutants are frequently selected in response to energy balance conditions, OP50 and BL21 may be ordinary examples of biofilm defective mutants among laboratory *E. coli* strains.

We found that motility was greatly enhanced in BW25113 *fimH::Km*, compared to original BW25113 (Fig. 1E). It has been shown that down-regulation of the expression of genes responsible for the formation of pili, including *fimH*, result in an upregulation of *fliC*, leading to enhanced motility (Leatham *et al*., 2005). The high motility of BW25113 *fimH::Km* is likely caused by anticorrelated control between *fimH* and *fliC*. Even in high-motility BW25113 *fimH::Km,* FimH is required for biofilm formation (Fig. 1A). The lack of motility enhancement in OP50 *fimH::Km* (Fig. 1E) is likely due to OP50’s non-functional flagella.

Our finding that OP50 is a biofilm formation-deficient mutant suggests that a genetic mechanism of biofilm formation is unlikely to be a direct cause of OP50 clogging in MFDs. Experimentally, autoaggregation has been studied by examining bacterial sedimentation in a culture solution (Trunk *et al.*, 2018). Autoaggregation has been shown to be promoted by Van der Waals forces between adhesion proteins, such as Flu/Ag43 (Lane *et al.*, 2007) and TibA (Hasman *et al*., 1999), and by depletion forces between bacterial cells that have been defined in colloid physics (Sherlock *et al*., 2005; Schwarz-Linek et al., 2010; Roux et al., 2005). Thus, it would be prudent to study how MFD clogging is affected by genetic and physical mechanisms of auto-aggregation in efforts to optimize conditions for using living bacteria for food in culture systems in MFDs.

## Methods

**Biofilm assay**Biofilm was quantified by a crystal violet (CV) (Tokyo Chemical Industry Co., LTD. Japan) quantitation method (Pratt and Kolter, 1998, Roux et al., 2005). A single *E. coli* strain colony was inoculated in 1 ml LB medium in a polystyrene 24-well plate (Multidish 24 wells Nunclon Delta SI, 142475, Nunc, Denmark, and 24-well Cell Culture Plate, 3524, Corning, USA) or glass tube (IWAKI 10X 100 mm 9832-1310 Asahi Glass Co. LTD., Japan), and cultured at room temperature for 2 d. After cultivation, the plate or glass tube was washed twice with 2 ml phosphate buffered saline and then incubated with 1% CV solution for 1 h. After CV staining, the plate or glass tube was washed with 2 ml phosphate buffered saline, and CV was resolved by 200 µl ethanol:acetone (8:2) solution. CV in ethanol:acetone solution was quantified by optical density at 600 nm (Microplate reader, Epoch2, BioTek, USA).

**Isolation of OP50 *fliC*::*Km* and OP50 *fimH::Km***Km cassettes, *fliC*::*Km* and *fimH::Km* in JW1908: BW25113 *fliC*::*Km* or JW4283: BW25113 *fimH::Km*, were transferred to OP50 by P1 transduction. The presence of *fliC::Km* and *fimH::Km* in OP50-derived colonies was checked by polymerase chain reaction (PCR) using a primer set between K1 or K2 primers (Datsenko and Wanner, 2000) and *fimH* confirming F primer (CACAATCAGCGCACTTCCCGTTACAG) or *fliC* confirming F primer (AGAAAAGAGTATTTCGGCGACTAAC) with bacteria picked up from a colony of candidates OP50 *fimH::Km* and OP50 *fliC*::*Km.* Bacteria obtained from all colonies of candidate OP50 *fimH::Km* and OP50 *fliC*::*Km* were subjected to PCR with the indicated primers; PCRs amplified DNA fragments of expected size (~700 base pairs) when a primer set containing K1 (but K2) primer was used. We used clone 1 for OP50 *fimH::Km* and OP50 *fliC*::*Km*.

**Motility assay**Motility of bacteria was compared by quantitation of colony size after culturing on LB plates with 0.3% agar (Eaves-Piles *et al*., 2008; Rashid and Kornberg, 2000). One-microliter aliquots of overnight culture solution of LB media were spotted onto soft agar LB plates and incubate for 2 d at room temperature.

**Statistical analysis**Statistical testing was performed with paired Student’s t-tests and p-values in multiple comparison were determined after Bonferroni correction. We considered p < 0.05 to be statistically significant.

## Reagents

BW25113 *fliC*::*Km* and JW4283: BW25113 *fimH::Km* were obtained from the National Institute of Genetics, Japan.
